# Morphological and molecular characterization of *Onchocerca fasciata* (Nematoda, Onchocercidae) from dromedary camels (*Camelus dromedarius*) in Iran

**DOI:** 10.1051/parasite/2018045

**Published:** 2018-09-20

**Authors:** Mohammad Mirzaei, Younes Ghahvei, Emilie Lefoulon, Riccardo Paolo Lia, Domenico Otranto, Coralie Martin, Alireza Sazmand

**Affiliations:** 1 Department of Pathobiology, Faculty of Veterinary Medicine, Shahid Bahonar University of Kerman Postal code: 7616914111 Kerman Iran; 2 Genome Biology Division, New England Biolabs, Inc. 240 County Rd Ipswich MA 01938 USA; 3 Dipartimento di Medicina Veterinaria, Università degli Studi di Bari Str. prov. per Casamassima km 3 70010 Valenzano Bari Italy; 4 Unité Molécules de Communication et Adaptation des Microorganismes, Muséum national d’Histoire naturelle, CNRS 75231 Paris cedex 05 France; 5 Department of Pathobiology, Faculty of Veterinary Science, Bu-Ali Sina University Postal code: 6517658978 Hamedan Iran

**Keywords:** *Onchocerca fasciata*, Vector-borne disease, Light microscopy, Phylogeny, dromedary

## Abstract

Skin nodules of *Onchocerca fasciata* Railliet and Henry, 1910 (Spirurida, Onchocercidae) are a common finding in dromedary camels, though with a minimal clinical impact. There is little information about the morphology, molecular make-up and pathological impact of this parasite. *Onchocerca fasciata* nodules (1.3–2.1 cm in diameter and 509–841 mg in weight) were detected on the neck region in 31.5% of dromedary camels examined in Kerman province, southeastern Iran. Of 38 isolated nodules, only 23 (60.5%) contained viable worms. Measurement and morphological analyses were performed on isolated female worms by light microscopy. The identification of *O. fasciata* specimens was confirmed by sequence analysis of two mitochondrial genes (12S rDNA and *cox*1), which showed 0.4% divergence from available *O. fasciata* sequences. In addition, a phylogeny of filarial nematodes was constructed, based on these two mitochondrial genes and five nuclear genes (18S rDNA, 28S rDNA, *MyoHC*, *rbp1*, *hsp70*); this indicated that *O. fasciata* belongs to clade ONC3 of Onchocercidae, with representatives of the genera *Onchocerca* and *Dirofilaria*. Within the genus *Onchocerca*, *O*. *fasciata* is grouped with bovine parasitic species and the human parasitic *Onchocerca volvulus*, which suggests an impact of domestication on the radiation of the genus. Data provided here on the distribution and morphology of *O. fasciata* contribute to the molecular identification and phylogenetic position of the species.

## Abstract (fr)

Les nodules cutanés d’*Onchocerca fasciata* Railliet and Henry, 1910 (Spirurida, Onchocercidae) sont fréquents chez les dromadaires, mais avec un impact clinique minime. Les informations sur la morphologie, la composition moléculaire et l’impact pathologique de ce parasite sont limitées. Des nodules d’*O. fasciata* (1.3–2.1 cm de diamètre pour un poids de 509–841 mg) ont été détectés dans le cou de 31.5 % des chameaux examinés dans la province de Kerman, en Iran. Sur 38 nodules isolés, seulement 23 (60.5 %) contenaient des vers viables. Des analyses morphologiques et des mesures ont été effectuées par microscopie optique sur des vers femelles isolées. L’identification des spécimens d’*O. fasciata* a été confirmée par l’analyse de deux gènes mitochondriaux (ADNr 12S et *cox1*) présentant une divergence de 0.4 % avec les séquences d’*O. fasciata* disponibles. En outre, une phylogénie des nématodes filariens a été construite sur la base de ces deux gènes mitochondriaux et de cinq gènes nucléaires (ADNr 18S, ADNr 28S, *MyoHC*, *rbp1*, *hsp70*). Cette analyse montre que *O. fasciata* appartient au clade ONC3 des Onchocercidae qui comprend des représentants des genres *Onchocerca* et *Dirofilaria*. Au sein du genre *Onchocerca*, *O. fasciata* est regroupé avec des espèces parasitaires bovines et le parasite humain *Onchocerca volvulus*, ce qui suggère un impact de la domestication sur le rayonnement du genre. Les données fournies ici sur la distribution et la morphologie de *O. fasciata* contribuent à l’identification moléculaire et la position phylogénétique de l’espèce.

**Abbreviations**
*cox*1: cytochrome oxidase subunit I;
rbp1: RNA polymerase II large subunit;
hsp70: heat shock protein;
myoHC: myosin heavy chain;
PBS: phosphate buffered saline solution;
ND5: NADH dehydrogenase subunits 5.

## Introduction

The genus *Onchocerca* Diesing, 1841 (Spirurida, Onchocercidae) is one of the most studied genera of the filarial nematodes, as it includes parasite species of veterinary and medical interest such as *Onchocerca volvulus* (Leuckart, 1893), the causative agent of human “river blindness” in tropical regions [25]. Moreover, there is an increasing number of cases of zoonotic *Onchocerca* infections caused by *Onchocerca gutturosa* Neumann, 1910, *Onchocerca cervicalis* Railliet and Henry, 1910, *Onchocerca reticulata* Diesing, 1841, *Onchocerca lupi* Rodonaja, 1967, *Onchocerca dewittei japonica* Uni, Bain and Takaoka, 2001 and *Onchocerca jakutensis* Gubanov, 1964 [27, 32, 45]. Despite the occasional human cases of infection, the above species most frequently infect cattle, horses, deer, boars, dogs and cats. However, an increase in number and range of zoonotic *Onchocerca* infections has been observed in recent years and remains unexplained [34], in part because the information on *Onchocerca* species in ungulate hosts, including camels, is still scarce.

With a global population of over 28 million, dromedary camels (*Camelus dromedarius*) are bred as multi-purpose animals in arid and semi-arid regions worldwide [17], mainly due to their extreme resistance to harsh environmental conditions. Currently, four filarial species are known to affect dromedary camels [41], namely, *Onchocerca fasciata* Railliet and Henry, 1910 and *Deraiophoronema evansi* (Lewis, 1882) (syn: *Deraiophoronema cameli* Romanovitch, 1916; *Dipetalonema evansi*), which are specific to camels; and *O. gutturosa* and *Onchocerca armillata* Railliet and Henry, 1909, which parasitize a broad range of ruminants, including camels. *Onchocerca gutturosa* are usually recovered from the fascia covering the ventral surface of the lamellar parts of the nuchal ligament in dromedaries as observed in Sudan [15, 21] and Australia [20], and *O. armillata* can be isolated from the aorta of dromedaries in Nigeria [42] and Sudan [3, 21]. *Onchocerca fasciata* forms nodules in the subcutaneous tissue and nuchal ligament that may sometimes be misdiagnosed as tubercle granulomas, resulting in unnecessary discarding of carcasses [12]. No treatment or specific control strategies have been suggested against *O. fasciata* infection. Conversely, *D. evansi* can induce clinical illness in infected animals (e.g., pulmonary distress, orchitis, aneurysm of the spermatic cord, arteriosclerosis, heart failure, nervous impairments and even death) in camel-rearing areas of the world [39].

*Onchocerca fasciata* infection has been reported from many countries in Asia (e.g., Iran, Saudi Arabia, Jordan) [1, 9, 41] and Africa (e.g., Egypt, Ethiopia and Kenya) [14, 26, 38]. Despite the wide geographical distribution of onchocercosis in dromedaries, the biology of this parasite has not been studied in detail, including the insect vector species. A few studies have been performed on the histochemical distribution of several hydrolytic enzymes and dehydrogenases in adult *O. fasciata* [30, 31] and fine structure of female specimens have been described using transmission electron microscope examination [13]. Similarly, the molecular characterization of filarial parasites of camel is scarce with only two publications including sequences of either *O. fasciata* [23] or *D. evansi* [40]. These phylogenies were based on only two or three mitochondrial genes and are characterized by unresolved topology, not clearly identifying relationships between the species. Recently, a larger phylogeny of filarial nematodes based on seven genes (mitochondrial and nuclear loci) was published, but neither *D. evansi* nor *O. fasciata* were included [24]. Here we present a reappraisal of the morphological and morphometric description of *O. fasciata* based on light microscopy and molecular characterization, as well as a phylogenetic analysis of filarial nematodes including *O. fasciata*.

## Materials and methods

### Ethics

Parasites used for this study were recovered from skin nodules collected from dromedary camels in slaughterhouses in accordance with the veterinary laws of I.R. Iran.

### Study area, isolation of worms and microscopic analyses

From April to May 2016, a total of 76 dromedary camels aging from 1 to 12 years and of both sexes (43 males and 33 females) were inspected for the presence of skin nodules in slaughterhouses of Kerman, southeastern Iran (30.2839° N, 57.0834° E). Nodules were recovered in accordance with the veterinary laws of I.R. Iran then transferred to the Faculty of Veterinary Medicine, Shahid Bahonar University of Kerman. Extra tissues surrounding the nodules were carefully removed and nodules digested by collagenase to isolate the adult worms from the nodules [28]. Briefly, nodules were incubated in phosphate buffered saline solution (PBS) containing type I collagenase (Sigma-Aldrich, USA) at a final concentration of 2.25 mg/mL for 24–48 h at 37 °C. The helminth mass and tissues were then washed with tap water and placed in petri dishes. Individual entire and broken worms were isolated under a dissecting microscope, sex was determined and each worms’ length was measured. Filariae were then fixed in 70% ethanol. Fixed worms were immersed in lactophenol solution to observe anatomical structures under light microscope. Mineralized worms were identified by obliteration of cuticular ridges and vertical annulations [18]. Measurements are provided as minimum-maximum, followed by mean in parentheses.

### Molecular procedures

One female was used for molecular analysis and accessioned as specimen 105YT in the collection of the National Museum of Natural History of Paris (MNHN), France. Genomic DNA was extracted with the QIAamp micro kit (Qiagen, Germany), according to the manufacturer’s instructions. A preliminary step of disruption for two cycles of 30 s each at 30 Hz using a TissueLyser II (Qiagen, Germany) was added. Seven partial sequences of seven genes were amplified according to Lefoulon et al. [24]: two mitochondrial genes (12S rDNA and cytochrome oxidase subunit I (*cox*1)) and five nuclear genes (18S rDNA, 28S rDNA, the myosin heavy chain (*MyoHC*), RNA polymerase II large subunit (*rbp1*), 70 kilodalton heat shock proteins (*hsp70*)). Polymerase chain reaction (PCR) products obtained were directly sequenced and the seven sequences were deposited in GenBank (http://www.ncbi.nlm.nih.gov/) under the accession numbers: MG188679 (12S rDNA), MG188678 (*cox*1), MG188681 (18S rDNA), MG188680 (28S rDNA), MG188684 (*MyoHC*), MG188683 (*rbp1*), and MG188682 (*hsp70*).

### Molecular identification and phylogenetic analysis

Two out of the seven sequenced genes were previously used for molecular identification: 12S rDNA and *cox*1 markers [16]. The produced sequences of *O. fasciata* and previously published sequences of *Onchocerca* species (Supplementary Table S1) were aligned using MAFFT [22]. The sequence divergence between *Onchocerca* species was estimated by the number of base differences per site between two sequences (*p*-distance) using MEGA5 [43].

Sequences of *O. fasciata* generated and previously published sequences (Supplementary Table S2) were independently aligned, trimmed and then concatenated. Two matrices were produced: a first matrix including all filarial nematodes and two outgroups to root the tree, *Filaria latala* Chabaud and Mohammad, 1989 (Spirurida, Filariidae) and *Protospirura muricola* Gedoelst, 1916 (Spirurida, Spiruridae); a second matrix including only *Onchocerca* species (Supplementary Table S3) was produced with *Dirofilaria* species as the outgroup to root the tree. JModelTest analysis [35] was performed to establish the evolution model best adapted to the sequence alignment for each individual gene using the corrected version of the Akaike Information Criterion (AIC; Supplementary Table S4). Phylogenetic relationships between onchocercid taxa, based on the concatenated dataset, were determined by Bayesian inference using MrBayes [37]. A partitioned model was implemented to estimate evolution parameters separately for each gene. Two runs were performed using five million steps with four chains, with tree sampling every 1000 generations; the first 1250 points were discarded as burn-in and posterior probabilities were calculated from these post-burning trees.

## Results

### Rate of infection and nodules' examination

Twenty-four out of 76 examined dromedary camels (31.5%) harbored single or multiple skin onchocercian nodules on the neck region. Infected camels were aged 1–7 years and were of both sexes (11 males and 13 females). Infected camels harbored mineralized-only (*n* = 6, 25%), viable-only (*n* = 11, 45.8%), or mixed nodules (*n* = 7, 29.2%). From 38 isolated nodules, 23 contained viable worms (60.5%). The size of mineralized and viable nodules was 1.5–2.1 (1.85) cm and 1.3–2 (1.52) cm, respectively. Weight of mineralized and viable nodules was 609–841 (755) mg and 509–822 (607) mg.

### Morphological description of female and microfilariae of *Onchocerca fasciata*

Twenty female worms were recovered from nodules, but no males were recovered.

**Figure 1.**
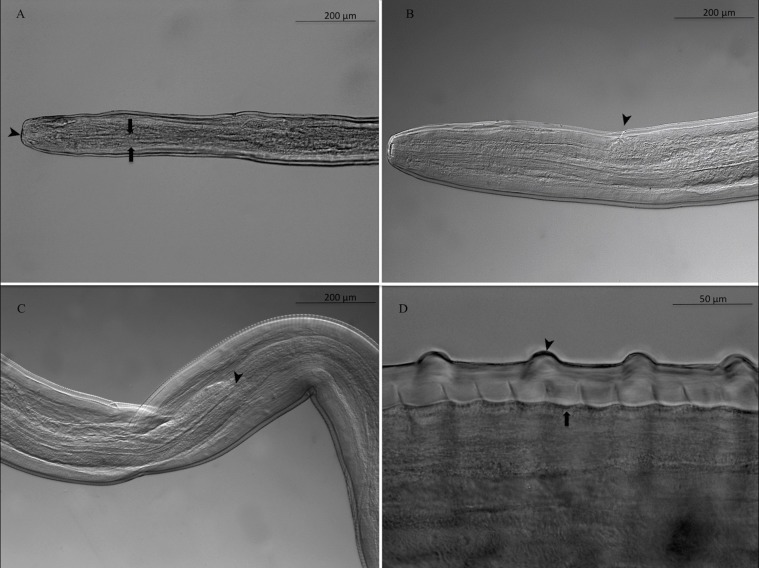
Adult female of *Onchocerca fasciata*. A. cephalic extremity in lateral view. The minute mouth orifice (arrowhead) and muscular esophagus (arrow). B. Vulvar opening (arrowhead). C. Esophago-intestinal junction (arrowhead). D. Mid-body region of the striated cuticle bearing annular transverse external ridges (arrowhead) and internal striae (arrow).

**Figure 2.**
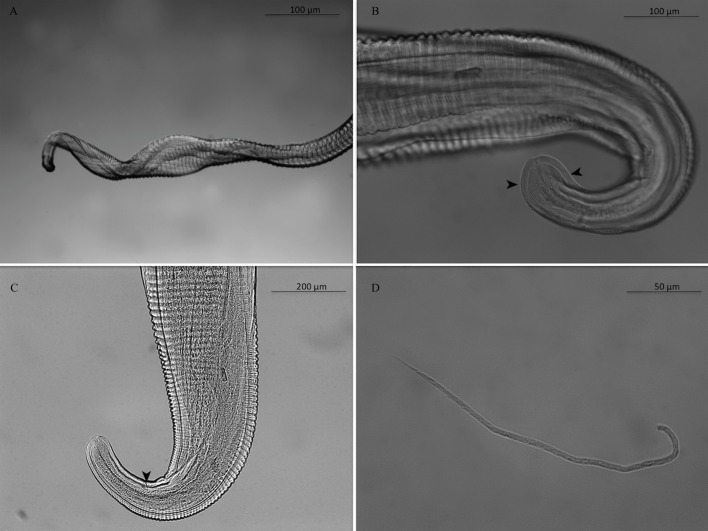
Adult female of *Onchocerca fasciata*. A. Posterior end with helicoidal twist. B. Posterior end rounded, with cuticle ridges becoming smooth (arrowhead). C. Tail, the anus (arrowhead). D. Microfilaria of *O. fasciata* isolated from the female uterus: anterior end rounded and tail pointed.

Females: 3 complete, 16 with one extremity only, 1 without extremities. Anterior end rounded, mouth orifice minute (Fig. 1A). Long muscular and glandular esophagus length 1940–2100 (2020) μm, width 102–104 (103) μm at the esophagus-intestinal junction level. Nerve ring at 212–230 (221) μm from the apex (Fig. 1A). Vulva 680–712 (696) μm from apex (Fig. 1B). Female body length 63–117 (90) cm, width 176–210 (196) μm at the esophagus-intestinal junction level (Fig. 1C). Cuticle 24–31 μm thick with two distinct layers (Fig. 1D). Presence of transverse external ridges (43 μm distant from each other at mid-body) and internal striae (21 μm between two striae at mid-body). Caudal end ventrally curved (Fig. 2A and Fig. 2B). Tail length 238–258 (248) μm (Fig. 2C).

Microfilariae from uteri (10 analyzed): body length 241–257 (251) μm, width 3.1–3.5 (3.3) μm; no sheath; blunt head; fine pointed tail (Fig. 2D).

### Molecular identification and phylogenetic analysis

The *cox*1 nucleotide mean divergence between the *O. fasciata* sequence and other *Onchocerca* sequences was 9.4% (minimum: 5.5% with *O. gutturosa* AJ271617; maximum: 12.2% with *O. ramachandrini* Bain, Wahl and Renz, 1993 KC167356) (Supplementary Table S1). The nucleotide sequence generated was 0.4% divergent from an unpublished sequence available in the NCBI database (JQ316672) indicated as belonging to *O. fasciata* species. The 12S rDNA mean divergence between the *O. fasciata* sequence and other *Onchocerca* sequences was 5.5% (minimum: 2% with *Onchocerca* sp. JX075209; maximum: 11.8% with *O. dewittei japonica* AM779816) (Supplementary Table S1). The produced sequence was 0.4% divergent from the previously published 12S rDNA sequence of *O. fasciata* collected from a dromedary camel in Saudi Arabia (DQ523744) [23].

**Figure 3.**
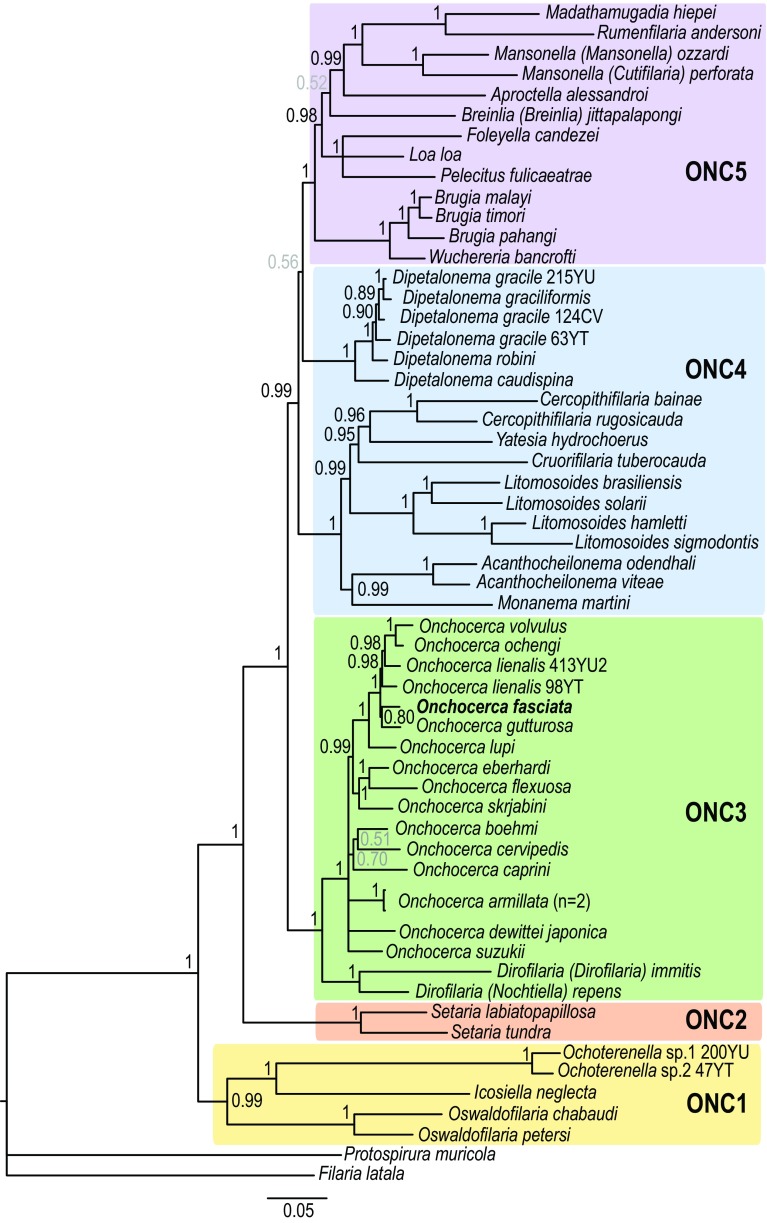
Phylogeny of filarial nematodes based on partitioned concatenated datasets of seven markers. Analysis is based on 12S rDNA, cytochrome oxidase subunit I (*cox*1), RNA polymerase II large subunit (*rbp1*), heat shock protein (*hsp70*), myosin heavy chain (*myoHC*), 18S rDNA and 28S rDNA sequences. *Filaria latala* and *Protospirura muricola* were used as outgroups. The topology was inferred using Bayesian inference. Nodes are associated with Bayesian posterior probabilities based on one run of five million generations. The onchocercid groups are indicated as ONC1 to ONC5 according to Lefoulon et al. [24]. Bold represents the newly sequenced specimens.

**Figure 4.**
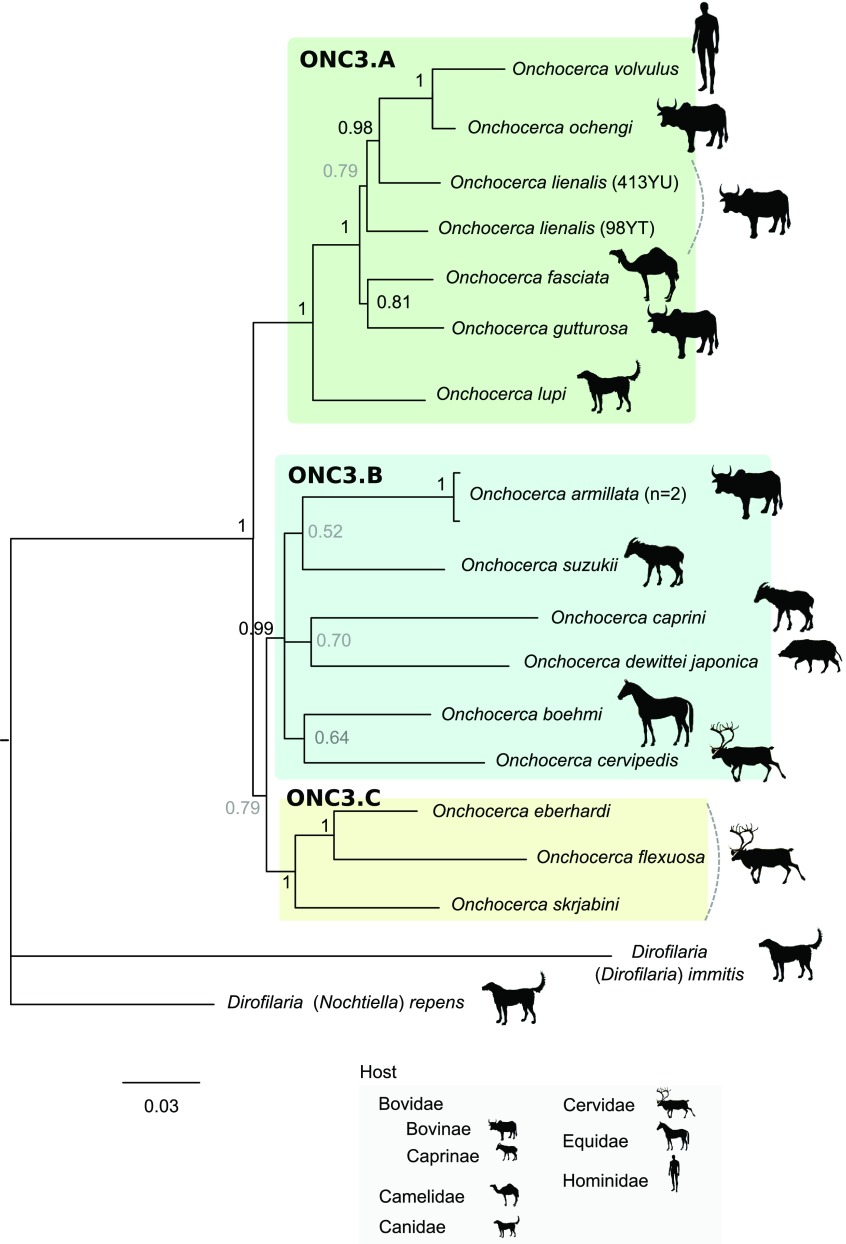
Phylogeny of the *Onchocerca* genus based on partitioned concatenated datasets of seven markers. Analysis is based on 12S rDNA, cytochrome oxidase subunit I (*cox*1), RNA polymerase II large subunit (*rbp1*), heat shock protein (*hsp70*), myosin heavy chain (*myoHC*), 18S rDNA and 28S rDNA sequences. *Dirofilaria immitis* and *Dirofilaria repens* were used as outgroups. The topology was inferred using Bayesian inference. Nodes are associated with Bayesian posterior probabilities based on one run of five million generations. The scale bar indicates the number of nucleotide substitutions. The host vertebrate family (or subfamily) for each filarial species is indicated using the specified symbols.

In order to facilitate the description of the tree topology, the clades will be referred to as ONC1 to ONC5, as in Lefoulon et al. [24] (Fig. 3), and letters were added for the ONC3 clade description (Fig. 4). The phylogenetic analysis based on multiple genes clusters *O. fasciata* into clade ONC3 which gather the other *Onchocerca* and *Dirofilaria* species (Fig. 3). Within the *Onchocerca* genus, *O. fasciata* forms clade ONC3A with the human parasite *O. volvulus*, *O. lupi* infecting carnivores, as well as *O. gutturosa*, *O. lienalis* Stiles, 1892, and *O. ochengi* Bwangamoi, 1969 infecting cattle (Fig. 4, Supplementary Table S3). The current analysis presents *O. fasciata* as a sister taxon of *O. gutturosa,* even though the robustness of this clade associated with a posterior probability is low.

## Discussion

The rate of infection in the present study (31.5%) was in the range of that previously reported from Iran [41] and from Saudi Arabia [9], but higher than that recorded in Jordan [1] (2%) and Egypt [15] (2.6%).

*Onchocerca fasciata* specimens causing nodules were studied for the first time in 1909 in two dromedary camels imported from Pakistan to Australia [19]. Nodules, in the present study, were found in subcutaneous connective tissue of the neck region. Similarly, dromedary onchocercal nodules have a fibrous capsule, are firm, not sensitive to the touch and localize mainly on the head, neck, two sides of the abdomen, shoulders, nuchal ligament, and the thigh region [2]. Histopathological examinations have revealed that multifocal granulomatous inflammatory reactions associated with the parasites are observed with thick fibrous walls, cellular infiltrate of lymphocytes, plasma cells, macrophages, multinucleated giant cells and eosinophils, associated with different degrees of coagulation necrosis and calcification around the parasites [2]. The sizes of nodules in our study varied in diameter from 1.3 to 2.1 cm, in agreement with previous findings [2, 18]. In particular, *O. fasciata* nodules increase with the age of dromedary camels [18] as they may contain live, degenerated or calcified worms [2]. In this study, 60.5% of worms in nodules were viable, a much higher prevalence compared to previous findings (28%–34% in Saudi Arabia) [18, 29]. A high degree of mineralization in *O. fasciata* compared to other *Onchocerca* spp. may be a likely reason for the lack of apparent disease in camels [18]. Diagnosis of camel onchocercosis is typically based on the presence of nodules and on the detection of microfilariae in skin snips. A high microfilariae concentration was noticed either in the head and neck region, or evenly distributed under the skin all over the body [11].

Morphological and morphometric characters of *O. fasciata* presented herein are consistent with those previously described [9, 11, 14]. *Onchocerca fasciata* was originally described in 1910 based on mid-body fragments of females, thus without extremities. The worms were recovered from a nodule extracted from the subcutaneous connective tissue of the head of a dromedary in Punjab, Pakistan [36]. Subsequent studies, from 1933 to 1938, provided morphological data on the cephalic and caudal extremities of *O. fasciata* specimens, although the description of males remained incomplete [4, 19]. Indeed, the finding of small numbers of *O. fasciata* male nematodes is most likely due to the fact that they roam in the conjunctiva and do not participate in the formation of fibrous tissues [19]. The morphological redescription of *O. fasciata* by Bain and Nasher [8] was the first to include a complete description of the male with two specimens harvested on the periphery of a nodule. This morphological analysis revealed a numerical reduction of the caudal papillae with a small grouping near the cloaca, the papillae of the head as in the infesting stage, a vulva close to the anterior extremity, and a powerful esophagus with thick glandular portion and narrow lateral chords. Female and microfilaria measurements, and morphological characters from the current study correspond to those described before [4, 8, 13, 36], clearly identifying the filariae as *O. fasciata*. The length of the esophagus in female worms in our study was 1940–2100 μm, within the range indicated by Bain and Nasher [8], but different from the single measurement of 1600 μm described by Henry and Masson [19] and Badanine [4]. Females in the genus *Onchocerca* have specialized cuticular architecture with ridges and striae. It is more or less complex depending on the *Onchocerca* species. For example, transverse ridges are absent in *O. suzukii* Yagi, Bain and Shoho, 1994 from bovids and *O. boehmi* (Supperer, 1953) from equids, and the internal striae are absent in *O. dewittei dewittei* Bain, Ramachandran, Petter and Mak, 1977, *O. ramachandrini*, and *O. dewittei japonica* from suids. On the other hand, the cuticle presents 3–4 striae between adjacent transverse ridges in *O. skrjabini* Ruchljadev, 1964, *O. garmsi* Bain and Schulz-Key, 1974, *O. jakutensis*, *O. alcis* Bain and Rehbinder, 1986 and *O. eberhardi* Uni and Bain, 2007 from cervids. *O. lupi* in carnivores has 2 striae between adjoining ridges. However, some variability has also been observed in domestic bovids with *O. gutturosa*, which has 4–7 striae per inter-ridge with sinuous ridges branched in lateral fields. Regarding *O. fasciata,* our measurements are smaller than those obtained by Bain and Nasher [5], with 43 μm instead of 75–80 μm measured between each ridge at mid-body of the worms. Such differences may be produced due to the development (length) of adult worms [44] and/or to a lack of precision of the mid-body position.

The molecular identification is concordant with the available sequences and published data [23, 47]. The molecular phylogeny places *O. fasciata* in the ONC3 clade of the Onchocercidae, which includes the *Onchocerca* and *Dirofilaria* species [24]. To date, only four sequences of *O. fasciata* from mitochondrial genes and one ribosomal region are available in the NCBI database: two available *cox*1 and ribosomal ITS region sequences in GenBank [46, 47] and three published by Krueger and colleagues [23]. The analyses of divergence for both *cox*1 and 12S rDNA sequences confirm the morphological identification. In addition, the observed high interspecific divergence validates the delineation of *O. fasciata* among the other *Onchocerca* species. As previously suggested, the *cox*1 analysis presents better discrimination between the species [16, 25].

Up to now, only one molecular phylogeny based on mitochondrial genes (12S rDNA, 16S rDNA and *ND5*) has included *O. fasciata* [23]. The species was described either as a sister taxon of *O. lienalis* or as a sister taxon of an *Onchocerca* species African clade including *O. volvulus*, *O. lienalis* and *O. dukei* Bain, Bussiéras and Amégée, 1974. However, this phylogeny was characterized by multifurcated branches, indicating unresolved topology. Indeed, the evolutionary relationships between *O. fasciata* and *O. gibsoni* (Cleland and Johnston, 1910), *O. gutturosa* or *O. jakutensis* remained unclear in Krueger and colleagues [23]. Our molecular analysis presents *O. fasciata* belonging to the clade ONC3A (Figure 4) which includes *O. lupi*, *O. gutturosa*, *O. lienalis*, *O. ochengi* and *O. volvulus*. The analysis of nuclear genes, in addition to mitochondrial genes, is more informative, and suggests that *O. fasciata* is closely related to *O. gutturosa* although robustness of this clade remains moderate. Although *O. gutturosa* primarily infects cattle, infections in camels have also been reported [15, 20, 21]. These results contradict the evolutionary hypotheses based on morphological traits.

Some anatomical characters, such as caudal papillae, cephalic papillae, development of esophagus, position of the vulva, and morphology of the cuticle of the female, have phyletic value for the *Onchocerca* genus [5, 7]. According to these characters, it was suggested that *O. fasciata* has morphological traits considered to be ancestral, such as the reduction in number and pattern of caudal papillae, an anterior position of vulva, a well-developed musculature of the female and a strongly divided oesophagus as seen in species infecting equids *O. reticulata* Diesing, 1841, *O. cervicalis* Railliet and Henry, 1910 and *O. raillietti* Bain et al., 1976 [8]. Although the latter species are not included in the sampling, the current molecular analysis suggests that *O. fasciata* species is not derived from an older independent speciation event. Nevertheless, *O. fasciata* has morphological traits that appear to be derived, such as the presence of transverse ridges in the female cuticle [8]. All *Onchocerca* species belonging to ONC3A clade present female cuticular architecture with ridges [25]. The structure of the female cuticle might have phyletic value for the *Onchocerca* genus, though larger sampling is needed to test this hypothesis.

Speciation events are influenced by multiple factors, such as host migration; thus the host distribution could be linked with *Onchocerca* speciation. It has previously been suggested that the emergence of the genus *Onchocerca* could have occurred in Africa back to the Pleistocene period with two independent speciation events, one in the forest and one from the savannah with a host switch to the human [6, 10]. However, our current analysis does not allow us to support or reject this hypothesis because of bias in the collection of analyzed samples. For example, four species out of six in the ONC3A clade were collected in the Afrotropical region (with the exception of *O. lupi* and *O. lienalis*) but the known distribution of these species is more extended. Only *O. ochengi* is strictly limited to the Afrotropical region; *O. volvulus* has also been described in the Neotropical region; infection by *O. gutturosa* is cosmopolitan [25] and infection by *O. fasciata* has been described in the Palearctic region.

Recently, it was suggested that domestication of bovines, dogs and cats may have contributed to host-switching events that led to speciation within the clade comprising *O. volvulus*, *O. ochengi*, *O. lienalis*, *O. gutturosa* and *O. lupi* (mainly composed of parasites of domestic animals or humans) [21]. At least one host-switching event between domestic bovine and canid/felid hosts and one event between domestic bovine hosts and humans may have occurred. Our molecular analysis highlights that the *O. fasciata* parasite of camels belongs to this clade (ONC3A), suggesting another host-switching event between domestic bovine and camelid hosts. Domestication of dromedaries occurred between 2000–1000 years ago [33], which supports the hypothesis of a recent speciation of *O. fasciata* among the *Onchocerca* genus. This further supports the hypothesis that domestication may have contributed to the speciation of the genus *Onchocerca*.

## Availability of data and materials

All data generated or analyzed during this study are included in this published article and its Additional files 1–4.

## Competing interests

The authors declare that they have no competing interests.

## Supplementary Material

All data generated or analyzed during this study are included in this published article and its Additional files 1–4. The Supplementary Material is available at https://www.parasite-journal.org/10.1051/parasite/2018045/olm.
